# Macular and choroidal thicknesses in a healthy Hispanic population evaluated by high-definition spectral-domain optical coherence tomography (SD-OCT)

**DOI:** 10.1186/s40942-020-00270-9

**Published:** 2020-12-07

**Authors:** Diana A. Cortés, Daniela Roca, Pedro Iván Navarro, Francisco J. Rodríguez

**Affiliations:** 1 Fundación Oftalmológica Nacional, Calle 50 # 13-50, Bogotá, Bogota, Colombia; 2 Escuela de Medicina y Ciencias de la Salud, Universidad del Rosario, Bogota, Colombia; 3 Asociación Médica de Los Andes, Bogota, Colombia

**Keywords:** Spectral domain optical coherence tomography, Macular thickness, Choroidal thickness, Hispanic population, Normal cut-off values

## Abstract

**Purpose:**

To report normal values of macular and choroidal thickness obtained from a healthy Hispanic population using Optovue (Optovue Inc, Freemont CA, USA) spectral domain optical coherence tomography (SD-OCT).

**Design:**

Observational, cross-sectional, correlation study.

**Methods:**

A total of 290 eyes (145 healthy subjects) were included; 69% of subjects were female. The median age was 39 ± 29 years (IQR), with a range between 18 and 89 years. The study sample was stratified into three age groups: Group 1, 18–40 years (50.3%), Group 2, 41–60 years (30.7%), and Group 3, older than 61 years (19%). Central macular, perifoveal (inner quadrants), and parafoveal (outer quadrants) thicknesses were estimated. In addition, central and peripheral choroidal thicknesses were estimated. Data analysis was performed to calculate the standardized mean difference according to the variance (Student’s *t*-test) and its differences with Epidat 4.1.

**Results:**

Median macular central thickness was 250 ±30 µm (IQR) with Optovue. Median central choroidal thickness was 263 ± 48 µm (IQR). Median central choroidal thickness was greater than mean peripheral thickness. Macular evaluation showed a statistically significant difference in central, perifoveal, and parafoveal thicknesses, with lower values being recorded for the study sample compared with the manufacturer’s data.

**Conclusions:**

SD-OCT has become a useful tool to obtain high-resolution images of the macula and choroid. This method allows precise assessment of the retinal and choroidal layers to diagnose and follow up posterior segment diseases. We are reporting normal cut-off values of macular and choroidal thicknesses in healthy Hispanic subjects evaluated with Optovue SD-OCT as new diagnostic normal parameters for research and clinical activities.

## Introduction

Since the introduction of Optical Coherence Tomography (OCT) in the 20th century, it has been possible to assess retinal and choroidal diseases more precisely. OCT is a non-invasive transpupillary method aimed with a laser system to obtain accurate measurements in vivo of the retina and choroid layers [1]. These devices use two different technologies for imaging, known as time domain (TD) and spectral domain (SD). Spectral Domain OCT, also known as Fourier-domain OCT, acquires images 100 times faster than TD technology, giving higher image resolution [2, 3].

One of the most significant contributions of OCT is the quantitative assessment of retinal and choroidal thickness [1]. Some reports suggest that these measurements vary according to age and ethnicity. Therefore, all these thickness measurements and their respective variations in data obtained through OCT must be standardized to demographic data from healthy subjects belonging to various age and ethnic groups [2]. Macular thickness variations are commonly seen in eyes with retinal diseases such as macular edema, age-related macular degeneration, diabetic retinopathy, vascular occlusions, uveitis, and macular atrophy [4–7].

Published evidence confirms that thinning or thickening of the macula is well correlated with visual function [8]. Achieving normal macular and choroidal thickness values provides a parameter to evaluate patients with posterior segment diseases and becomes a benchmark for clinical and research activities. However, there is limited information from different ethnic groups, especially the Hispanic population, from which to obtain a reliable parameter to compare findings across Latin America with confidence [9–13]. Current clinical practice demands knowledge of normal values of macular and choroidal thickness in the Hispanic population for comparison with normal cut-off values included by manufacturers as a reference in technology manuals. Current normal SD-OCT cut-off values in Optovue have been developed by including data from different ethnic groups, with a small proportion of the sample representing the Hispanic population, which could induce a classification bias for this ethnic group. Of the 480 subjects enrolled in Optovue’s normal cut-off value study, 33% were Caucasian, 22% Asian, 20% African-American, 12% Hispanic, 12% Indian, and 1% comprised other ethnic groups [14]. Therefore, due to the absence of valid information regarding macular and choroidal thicknesses in the Hispanic population, our study aims to obtain this information from subjects evaluated at the Fundación Oftalmológica Nacional, in Bogotá Colombia.

## Methods

A cross-sectional and correlation study was carried out including 290 eyes from 145 healthy Hispanic subjects that were evaluated at the Fundación Oftalmológica Nacional in Bogotá, Colombia. The global median (± IQR) age was 39 ± 29 and 69% were female (Table 1). All subjects underwent a complete ophthalmological exam, including refractive error determined by an autorefractor. OCT scans were performed with Optovue. The obtained values were compared with the manufacturer’s normal values.

**Table 1:** Demographic characteristics of the study subjects

	Global	Female	Male
290 eyes	290	200	90 eyes
145 subjects	100%	69%	39%
Median age	39 ± 29 (IQR)	42 ± 34 years	44 ± 18 years

The ethnic category, Hispanics, as defined by the Office of Management and Budget (OMB) in 1978, refers to persons or descendants of people from Latin American countries or other Spanish cultures. Under this definition Hispanics are culturally and genetically a heterogeneous group. In Latin America, each country has its own demographic and genetic structure, with its own distinct migration history between regions. All Hispanics are basically trihybrid, their ancestral populations being European, African, and Native American [15]. For this study we included Hispanics who had at least two generations of Hispanic ancestors.

Healthy subjects were included who met the following criteria: age greater than 18 years, written informed consent to participate in the study, and visual acuity of 20/20 in all eyes included in the study. The exclusion criteria were myopia greater than − 5.00D, hyperopia greater than + 5.00D, diagnosis of glaucoma, history of eye diseases (retinal detachment, age-related macular degeneration, history of venous or arterial occlusions, retinal dystrophies, central serous chorioretinopathy, uveitis, intraocular tumors), systemic diseases (diabetes mellitus, high blood pressure), history of eye surgery (vitreoretinal surgery, intravitreal injections, complicated cataract surgery), presence of degenerative neurological diseases, and poor image quality taken by the Optovue.

Images were obtained using six radial macular probes centered on the fovea with equal angular distance and 20 tracking lines spaced by 200 µm, to achieve an axial and transverse resolution of 7–10 µm, respectively. The cross-sectional images were analyzed with a software program that automatically performs segmentation of the two edges on each OCT scan, one at the vitreoretinal interface and the other one in the retinal pigment epithelium (RPE), Bruch’s membrane complex.

### Study sample

Simple randomized sampling was conducted for gender and age. The study sample was stratified into three groups by age: Group 1 from 18 to 40 years old, Group 2 from 41 to 60, and Group 3, which included subjects older than 61 years. Sample size was calculated assuming a 50% proportion of normal patients attending the comprehensive eye clinic at Fundación Oftalmológica Nacional with a 95% confidence level and an absolute precision of 5%, reaching an estimated sample of 369 eyes. The total number of eyes included in the study was 290.

### Data analysis

A univariate analysis was performed for quantitative variables such as age and retinal and choroid thicknesses. A global analysis was performed and stratified by gender and age using a Student’s *t*-test for a standardized mean difference according to variance. The Epidat 4.1 statistical package was used for analysis.

### Ethical considerations

The study was conducted in accordance with the tenets of the Declaration of Helsinki and National regulations. All patients signed a statement of informed consent before enrollment, and all procedures were reviewed and approved by the appropriate institutional review boards and ethics committees.

### Global results

**Fig. 1:**
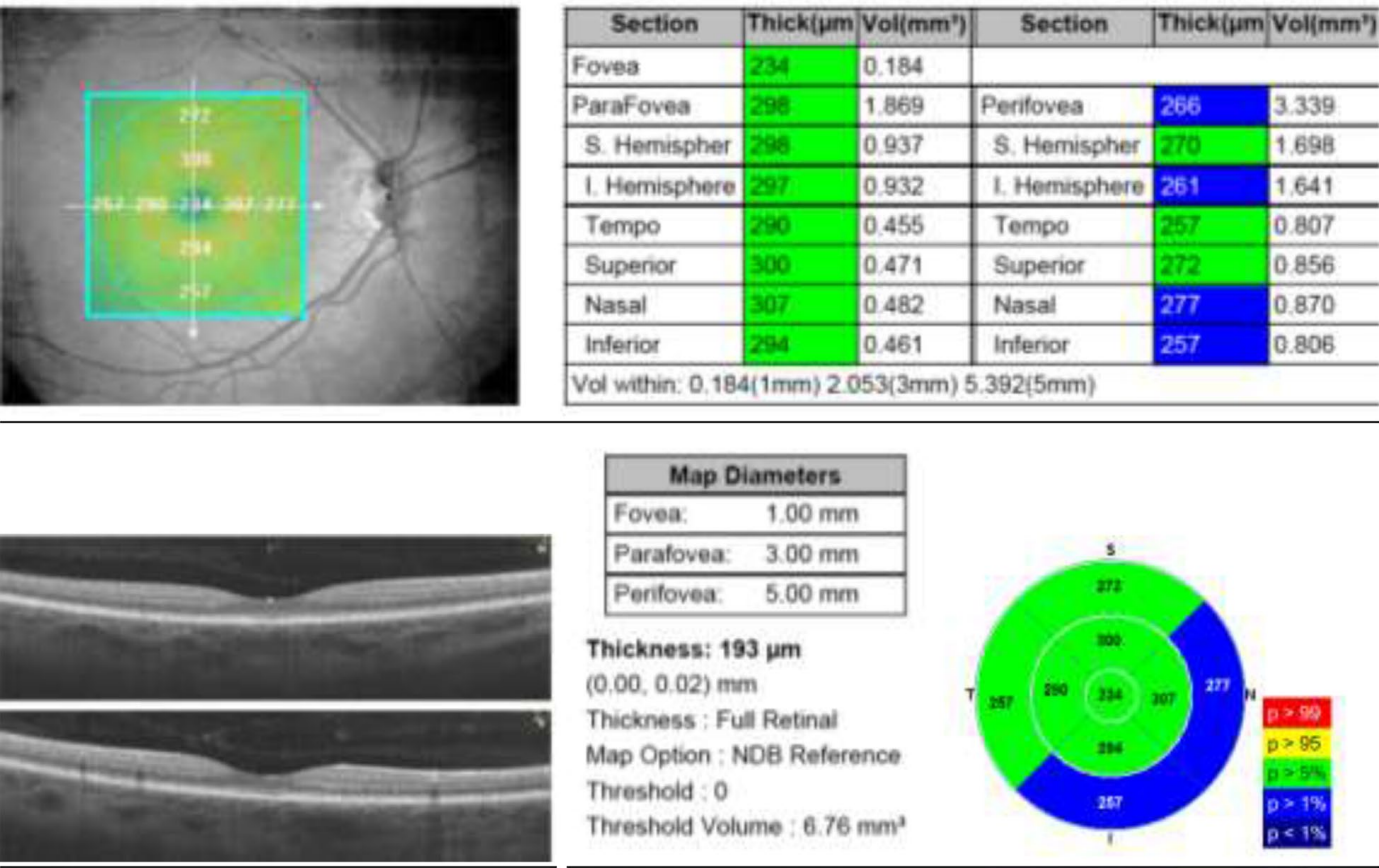
Macular thickness map using ETDRS circles of 1 mm, 3 mm, and 5 mm showing the mean thickness in each of the 9 subfields

Two hundred ninety eyes with a median (± IQR) age of 39 ± 29 were analyzed. Of them, 69% were women (220 eyes) with a median (± IQR) age of 42 ± 34 and 31% were men (90 eyes) with a median (± IQR) age of 44 ± 25. The age range of the sample was between 18 and 89 years. The sample was stratified into three age groups: Group 1, 18–40 years (50.3%), Group 2, 41–60 years (30.7%), and Group 3, older than 61 years (19%). The central macular, perifoveal (internal quadrants), parafoveal (external quadrants), central choroidal, and peripheral choroidal thicknesses were measured (Fig. 1).

**Fig. 2:**
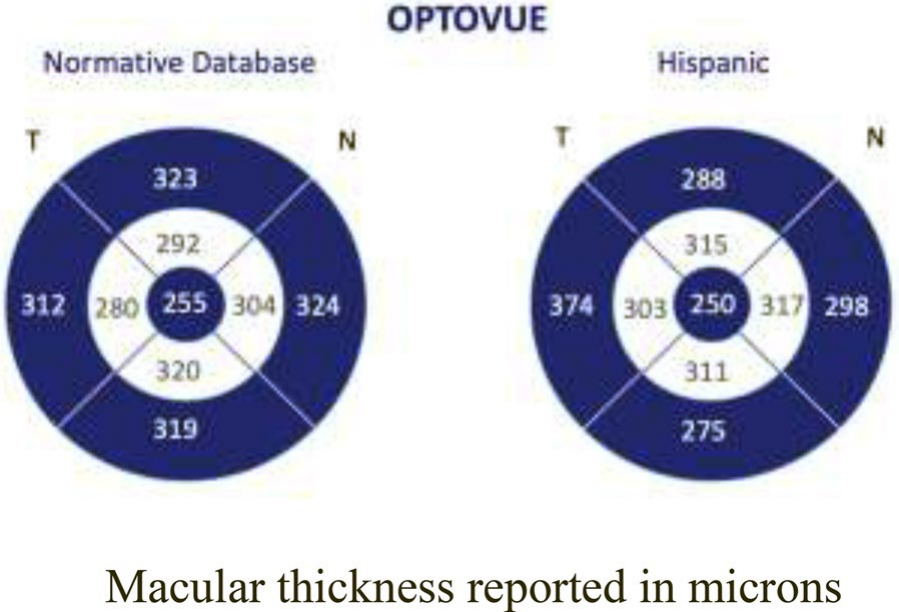
Central, perifoveal, and parafoveal macular thickness reported by the manufacturer and the normative basis of Hispanic subjects. Macular thickness reported in microns

The average central macular thickness obtained was 250 ± 30 µm, in contrast to the normal cut-off value reported by the manufacturer of 255 ± 22 µm. A thinner value (5 µm) was found in our study sample. An important difference was found in relation to the normative database reported by the manufacturer in the perifoveal measurements (inner macular) evaluated with Optovue. With this equipment, the following normal values were found: inner superior macular thickness: 315 ± 19 µm; inner inferior macular thickness: 311 ± 15 µm; inner nasal macular thickness: 317 ± 19 µm; inner temporal macular thickness: 303 ± 23 µm (Fig. 2).

Thinner parafoveal areas (external macular) were observed in the study sample. To assess whether or not there was a difference between the normal reported values for the equipment and the studied population, a standardized difference of means was used, for a confidence level of 95% using the Student’s *t*-test.

Statistically significant differences were found in central macular thickness, internal macular thickness (perifoveal), and external macular thickness (parafoveal), with the exception of the external nasal macular thickness, between the normative database reported by the Optovue manufacturer and the population studied.

The central retina was significantly thinner in the study sample, with a standard mean deviation (SMD) of 5.59 µm (95%CI 2.01–9.13, p < 0.002). Internal superior macular thickness showed a statistically significant difference of 7.37 µm (SMD, 95%CI 4.60–10.13 p < 0.000), being thinner in study sample. Inner inferior macular thickness showed a statistically significant difference of 7.67 µm (SMD, CI: 95% 5.18–10.15 p < 0.001), being thinner in the study sample. Inner nasal macular thickness showed a statistically significant difference of 7.78 µm (SMD, 95%CI (4.95–10.60, p < 0.001), being thinner in the study sample. Inner temporal macular thickness showed a statistically significant difference of 7.65 µm (SMD, 95%CI 4.81–10.48 p < 0.000), being thinner in the study sample.

A statistically significant difference was observed in outer superior macular thickness (SD: 3.49 µm, 95%CI: 1.01–5.96, p < 0.006), being thinner in the studied population compared with the normative database. Outer inferior macular thickness was found to be thinner in the studied population (SD: 44.40 µm, 95%CI: 41.62–47.18, p < 0.000). Outer temporal macular thickness had a statistically significant difference (SD: 5.12 µm, 95%CI: 2.24–7.99, p < 0.001), being thinner in the study sample compared with normal cut-off values.

### Results stratified by age

**Fig. 3:**
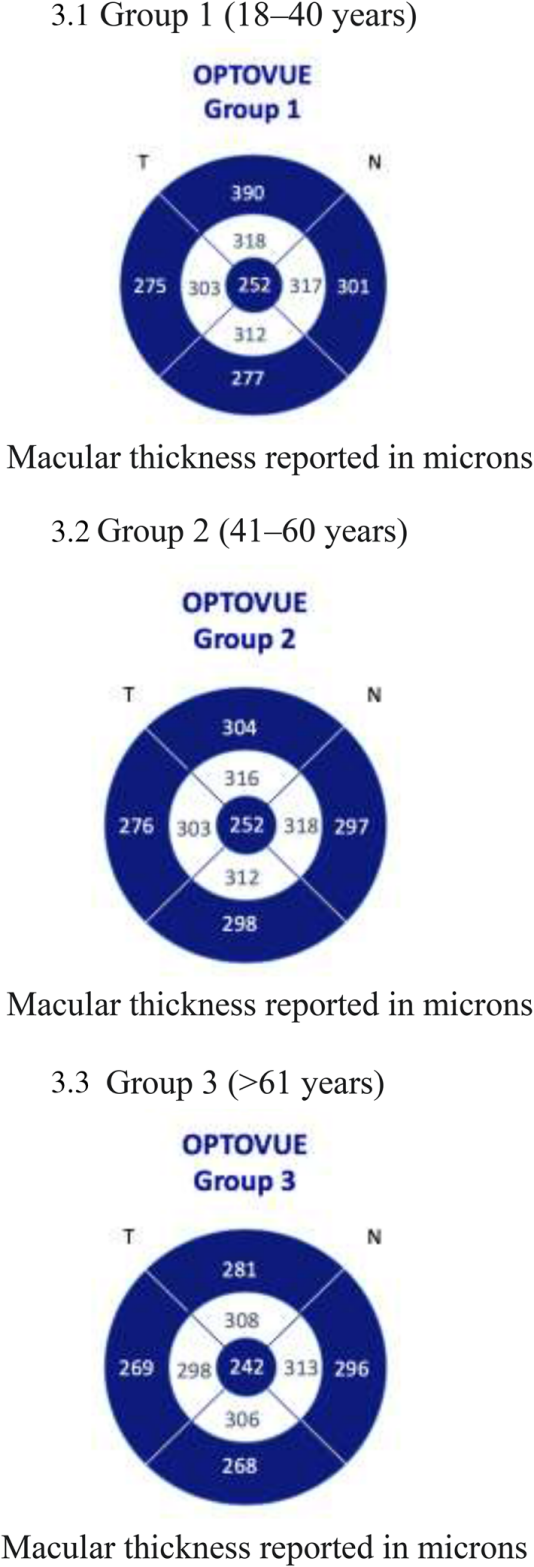
Central, perifoveal, and parafoveal macular thickness stratified by age group

The study sample was stratified into three age groups. Macular thickness showed variability when all groups were compared. Group 1 (age range 18–40 years) had greater macular thickness values at the central, perifoveal, and parafoveal levels than Group 2 (age range 41 to 60) and Group 3 (age range: older than 61 years). Group 2 had greater macular thickness values at the central, perifoveal, and parafoveal levels than Group 3 (age range: older than 60 years), evidencing a decrease in macular thickness in elderly patients (Fig. 3).

#### Group 1

We included 152 eyes (69.74% from female subjects) with a median (± interquartile range, IQR) age of 29 ± 11 years. The median (± IQR) central macular thickness was 252 ± 32 µm. Regarding inner macular areas, the results showed an inner superior with a mean (± SD) thickness of 318 µm ± 19 µm, an inner inferior with a mean (± SD) thickness of 312 ± 15.41 µm, an inner nasal area with a mean (± SD) thickness of 317 ± 19 µm, and an inner temporal area with a median (± IQR) thickness of 303 ± 23 um. Regarding the outer areas, outer superior evaluation showed a median (± IQR) thickness of 290 ± 22 um, outer inferior a median (± IQR) thickness of 277 ± 23 um, outer nasal a median (± IQR) macular thickness of 301 ± 20 um, and outer temporal macular evaluation showed a median (± IQR) thickness of 275 ± 23 µm (Fig. 3.1).

#### Group 2

We included 83 eyes (71.08% from female subjects) with a median (± IQR) age of 53 ± 8 years. Central macular thickness showed a median (± IQR) thickness of 252 ± 36 um. Regarding the inner macular areas, the inner superior showed a mean (± SD) thickness of 316 ± 1 um, the inner inferior a mean (± SD) thickness of 313 ± 15 um, the inner nasal a mean (± SD) thickness of 318 ± 15.52 um, and the inner temporal a median (± IQR) thickness of 303 ± 23 um. Regarding the outer macular areas, the outer superior showed a median (± IQR) thickness of 304 ± 22 um, the outer inferior a median (± IQR) thickness of 289 ± 16 um, the outer nasal a median (± IQR) thickness of 297 ± 19, and the outer temporal a median (± IQR) thickness of 276 ± 15 µm (Fig. 3.2).

#### Group 3

We included 55 eyes (63.64% from female subjects) with a median (± IQR) age of 65 ± 5 years. Central macular thickness showed a median (± IQR) value of 242 ± 28 um. Regarding inner macular areas, the inner superior showed a mean (± SD) thickness of 308 ± 19 um, the inner inferior a mean (± SD) thickness of 306 ± 15 um, the inner nasal a mean (± SD) thickness of 313 ± 19 um, and the inner temporal a median (± IQR) thickness of 298 ± 27 um. Regarding outer areas, the outer superior showed a median (± IQR) thickness of 281 ± 22 um, the outer inferior a median (± IQR) thickness of 268 ± 18 um, the outer nasal a median (± IQR) thickness of 296 ± 26 µm, and the outer temporal showed a median thickness of 269 ± 22 µm (IQR) (Fig. 3.3).

### Results stratified by gender

Two hundred eyes from female subjects (68.97% n = 290) were included with a median (± IQR) age of 39 ± 29 years. Retinal thickness areas showed a median (± IQR) central thickness of 248 ± 31 µm. Regarding inner macular areas, the inner superior showed a mean (± SD) thickness of 313 ± 18 µm, the inner inferior a mean thickness of 308 ± 14 µm (SD), the inner nasal a mean thickness of 313 ± 18 µm (SD), and the inner temporal a median (± IQR) thickness of 299 ± 22 um. Regarding outer macular areas, the outer superior showed a median (± IQR) thickness of 286 ± 19 um, the outer inferior a median (± IQR) thickness of 275 ± 20 um, the outer nasal macular a median (± IQR) thickness of 273 ± 18 um, and the outer temporal a median (± IQR) thickness of 296 ± 19 µm (Fig. 4.1).

No statistically significant difference was found in central macular thickness between the normal database from the manufacturer Optovue and the Hispanic study sample in the female group. In a comparison of inner macular areas, the inner superior SMD was 8.08 µm (95%CI: 4.46 − 11.69, p < 0.000), the inner inferior SMD 1.21 µm (95%CI: 4.36–10.45, p < 0.000), the inner nasal SMD 7.67 µm (95%CI: 4.05–11.28, p < 0.000), and the inner temporal SMD 7.67 µm (95%CI: 4.02–11.31, p < 0.000). These inner retinal area thicknesses in the Hispanic study sample were thinner than the normal values provided by the manufacturer. Regarding outer macular areas, the outer superior SMD was 5.29 µm (95%CI: 2.13–8.44, p < 0,001), the outer inferior SMD 6.20 µm (95%CI: 2.96–9.43, p < 0.000), the outer nasal SMD 7.71 (95%CI: 4.57–10.85, p < 0.000), and the outer temporal SD 6.29 µm (95%CI: 2.54–10.03, p < 0.001). The outer macula were significantly thinner in the Hispanic study sample compared with the normal value data provided by the manufacturer.

Ninety eyes from male subjects (31.03%, n = 290) were included with a median (± IQR) age of 44 ± 18 years. Retinal area thicknesses showed a median (± IQR) central thickness of 268 ± 28 um. Regarding inner macular areas, the inner superior showed a median (± IQR) thickness of 344 ±23 um, the inner inferior a mean (± SD) thickness of 340 ±15 um, the inner nasal a mean (± SD) thickness of 347 ± 17 um, and the inner temporal a mean (± SD) thickness of 333 ± 16 um. Regarding outer retinal areas, the outer superior showed a median (± IQR) thickness of 304 ± 20 um, the outer inferior a median (± IQR) thickness of 287 ± 21 um, the outer nasal a mean (SD) thickness of 314 ± 18 um, and the outer temporal a median (IQR) thickness of 290 ± 19 µm (Fig. 4.2). There was no statistically significant difference between the normal value database from Optovue technology and our Hispanic study sample.

**Fig. 4:**
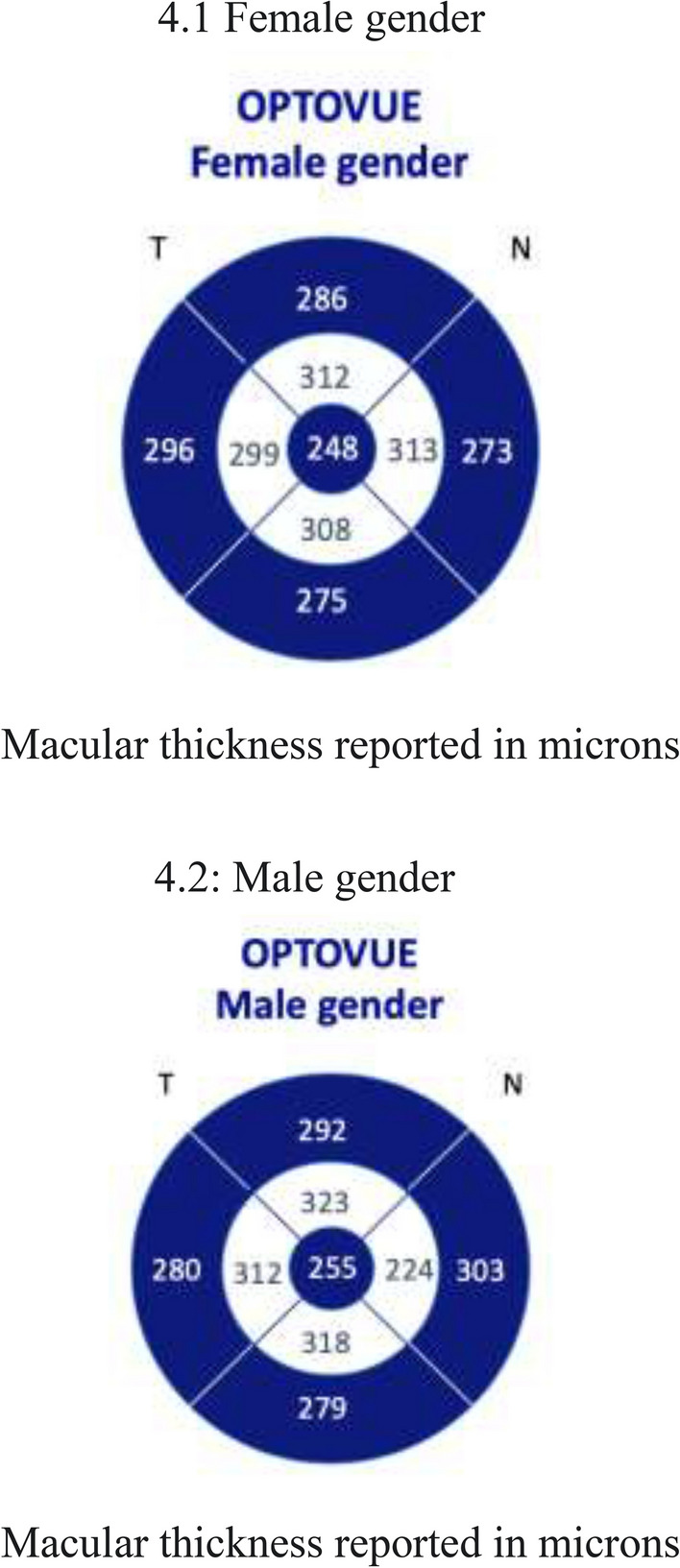
Central, perifoveal, and parafoveal macular thicknesses stratified by gender

### Choroid thickness

There were no previous publications regarding normal choroidal thickness values from Optovue technology. In our healthy Hispanic study sample, the median (± IQR) central choroidal thickness found with Optovue was 263 ± 48 um.

### Global results

We also measured paracentral choroidal thickness at 500, 1000, and 2000 µm from the center. Regarding paracentral nasal choroidal areas at 500, 1000, and 2000 um, we found a median (± IQR) thickness of 236 ± 64 um, 254 ± 46 um, and 237 ± 56 um, respectively. Regarding paracentral temporal areas, Optovue results showed a median (± IQR) choroidal thickness of 256 ± 47 um, 256 ± 49 um, and 234 ± 51 um, respectively (Fig. 5.1, 2).

**Fig. 5:**
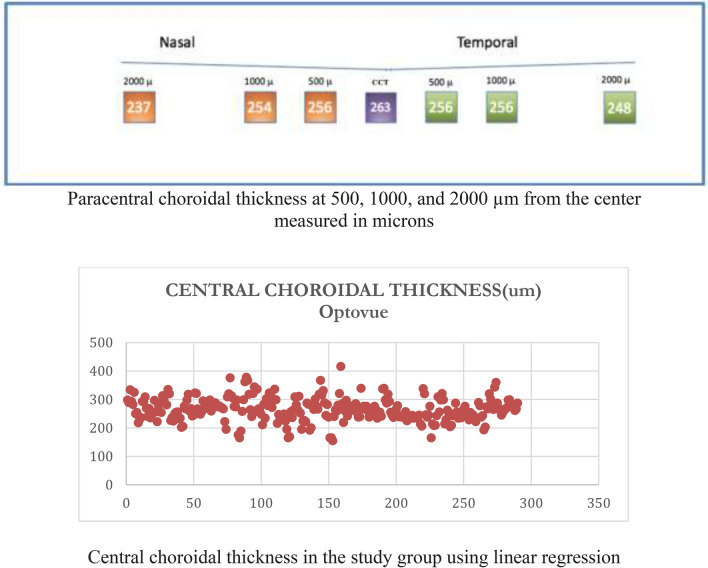
**5.1.** Optovue choroidal thickness (Global Analysis) , **5.2.** Optovue Central Choroidal Thickness (Global Analysis)

### Choroid thickness stratified by age

#### Group 1

**Fig. 6:**
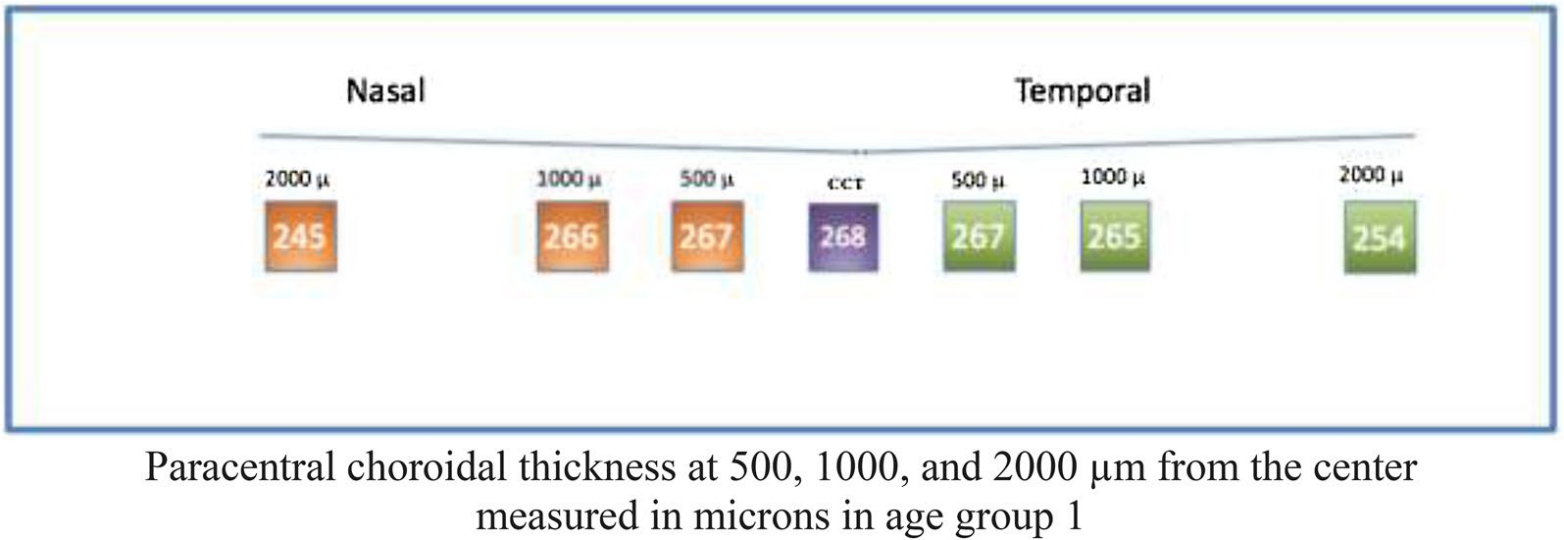
Optovue choroidal thickness by Age (Group 1)

Analysis of Group 1 showed a central median (± IQR) choroidal thickness of 268 ± 54 µm. Regarding paracentral nasal choroidal areas (500, 1000, and 2000 µm), assessment showed a median thickness of 267 ± 56 µm, 266 ± 57 µm, and 245 ± 54 µm (IQR), respectively. Regarding temporal areas (500, 1000, and 2000 µm), the median values found were 267 ± 50 um, 265 ± 47 µm, and 254 ± 46 µm (IQR), respectively (Fig. 6).

#### Group 2

**Fig. 7:**
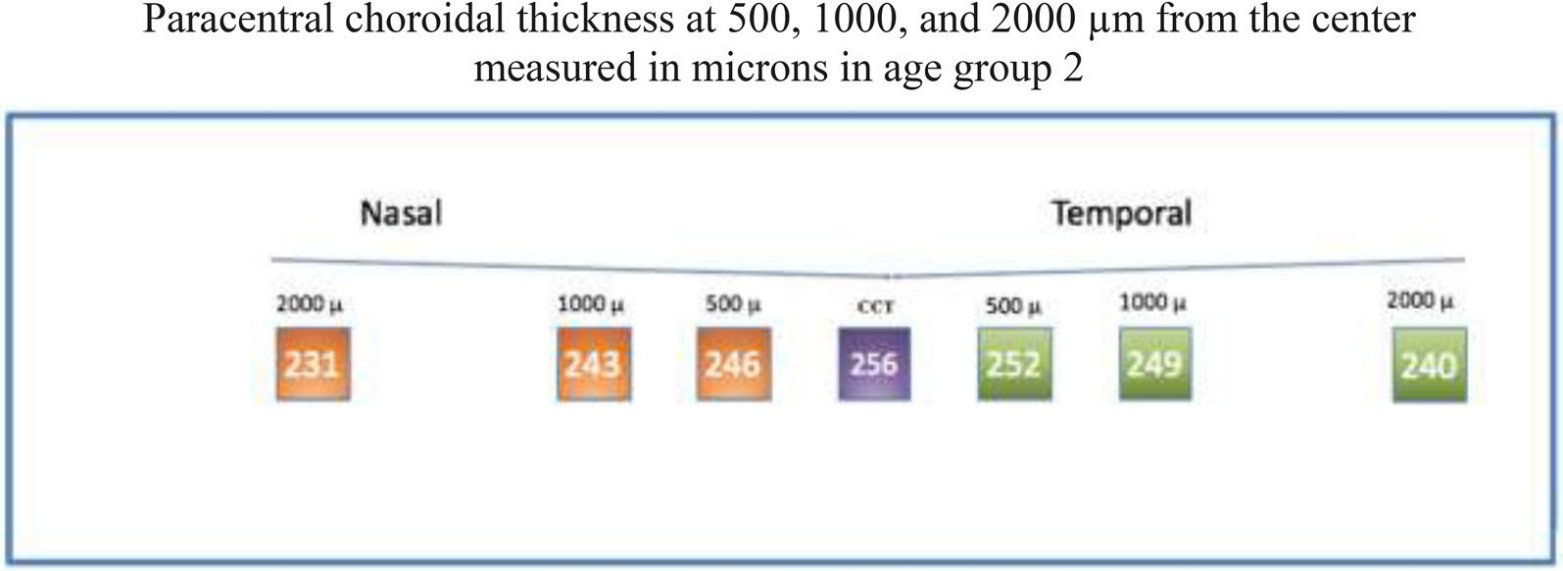
Optovue choroidal thickness by age (Group 2)

Analysis of Group 2 showed a median (± IQR) central thickness of 256 ± 41 µm. Regarding paracentral nasal choroidal areas (500, 1000, and 2000 µm), assessment showed a median (± IQR) thickness of 246 ± 47 µm, 243 ± 45 µm, and 231 ± 61 um, respectively. Regarding paracentral temporal areas, assessment showed a median (± IQR) thickness of 252 ± 56 um, 249 ± 30, and 240 ± 39 um, respectively (Fig. 7).

#### Group 3

**Fig. 8:**
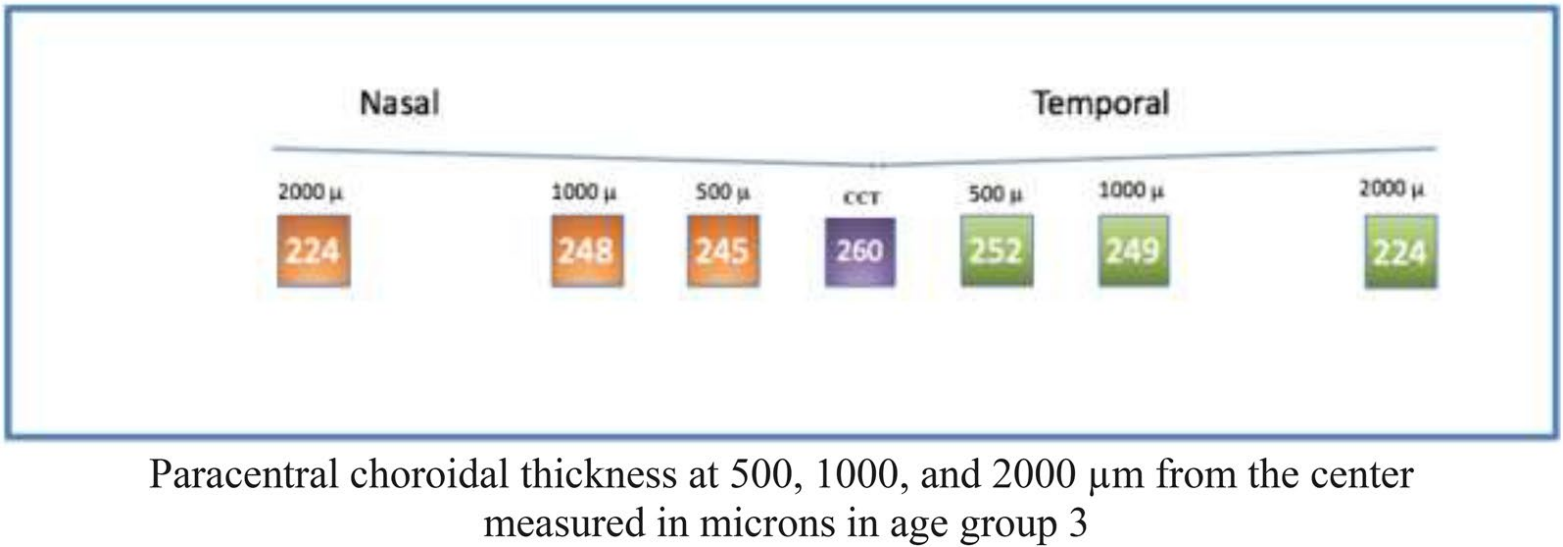
Optovue choroidal thickness by age (Group 3)

Analysis of Group 3 showed a median (± IQR) central thickness of 260 ± 43 µm. Regarding paracentral nasal choroidal areas (500, 1000, and 2000 um), assessment showed a median (± IQR) thickness of 245 ± 49 um, 248 ± 52 µm, and 224 ± 65 um, respectively. Regarding paracentral temporal choroidal areas (500, 1000, and 2000 µm), assessment showed a median (± IQR) thickness of 252 ± 56 um, 249 ± 39 um, and 224 ± 65 µm, respectively (Fig. 8).

### Choroid thickness by gender

#### Female sample

**Fig. 9:**
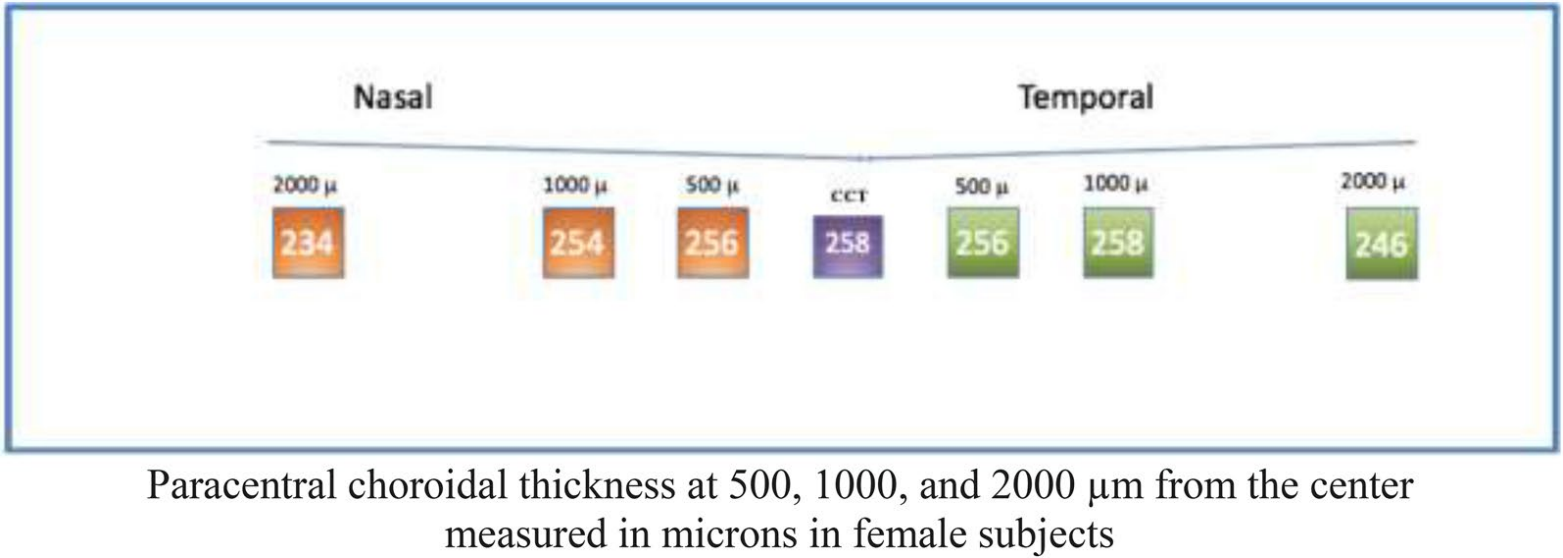
Optovue choroidal thickness (Female Subjects)

Analysis of female subjects showed a median (± IQR) central thickness of 258 ± 46 um. Regarding paracentral nasal choroidal areas (500, 1000, and 2000 um), assessment showed a median (± IQR) thickness of 256 ± 49 um, 254 ± 47 um, and 234 ± 55 µm (IQR), respectively. Regarding paracentral choroidal temporal areas (500, 1000, and 2000 um), assessment showed a median (± IQR) thickness of 256 ± 44 um, 258 ± 52 um, and 246 ± 46 um, respectively (Fig. 9).

#### Male sample

**Fig. 10:**
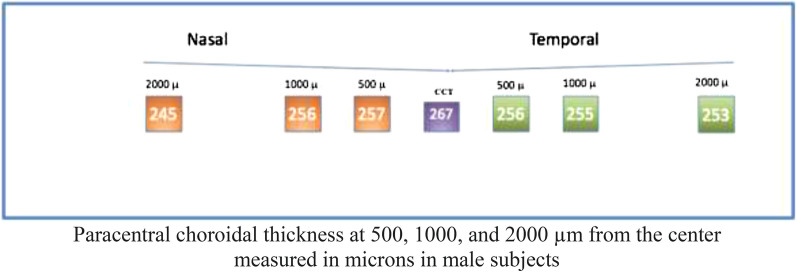
Optovue choroidal thickness (Male Subjects)

Analysis of male subjects showed a median (± IQR) central thickness of 267 ± 56 um. Regarding paracentral nasal choroidal areas (500, 1000, and 2000 um), assessment showed a median (± IQR) thickness of 257 ± 52 um, 256 ± 43 um, and 245 ± 54 um, respectively. Regarding paracentral temporal choroidal areas (500, 1000, and 2000 um), assessment showed a median (± IQR) thickness of 256 ± 53 um, 255 ± 43 um, and 253 ± 40 um, respectively (Fig. 10).

## Discussion

In recent years, spectral domain optical coherence tomography (SD-OCT) has become a useful tool that provides high-resolution images and valuable information in different diseases of the retina, choroid, and optic nerve. SD-OCT has revolutionized the diagnosis of several vitreoretinal diseases such as age-related macular degeneration, diabetic retinopathy, and macular diseases associated with edema due to different etiologies.

Since morphological characteristics can be seen in detail with this technology, it is necessary to establish normal cut-off values for macular and choroid thicknesses for clinical applications. While there are several studies that have reported them, there are no data in a healthy Hispanic population. Using new normal cut-off thickness values allows more accurate classification of healthy and diseased subjects in clinical and research scenarios. Thickness values obtained with Optovue in the study sample were statistically significantly different compared with the normal thickness value database reported by the manufacturer in central, perifoveal, and parafoveal thicknesses, except for the outer nasal macular area, most measurements being thinner in the Hispanic study sample. Gupta et al., in their Singapore Chinese Eye Study, measured macular thickness with SD-OCT confirmed that central macular area was the thinnest (250.38 ± 20.58 µm) and inner regions the thickest (319.33 ± 14.40 µm). They also concluded that retinal thickness decreases as it moves away from the fovea to periphery (276.67 ± 11.94 µm) [16]. Subjects included in Group 3 (> 60 years) were less than subjects included in Groups 1 and 2 and showed thinner retinal thickness values in all areas with progressive thinning in retinal layers with age. The central macula was the thinnest area and decreased progressively in the perifoveal and parafoveal areas according to a normal anatomical distribution. This finding was reported previously by Appukuttan et al. [2] and Grover et al. [3] in Indian and Caucasian populations, respectively. Due to the absence of a normal value database stratified by age from the manufacturer, we could not compare our results in age Groups 1, 2, and 3 to either confirm or dismiss differences in macular thickness. Our study, like others before it, found that gender and age are factors that influence macular thickness [16]. Adhi et al. reported that in Pakistan, mean foveal thickness in healthy individuals was 229 ± 20.46 µm and concluded that macular thickness varied depending on gender. Their result for central macular thickness was 266 ± 14.20 µm in males and 258.21 ± 10.03 µm in the female population. However, they did not find a statistically significant difference regarding age subgroup analysis [17].

Regarding the choroid layer, there are no previous reports regarding normal cut-off thickness values in the Hispanic population. Normal choroid thicknesses were described by Margolis et al. [18] using Spectralis (Heidelberg Engineering) and by Manjunath et al. using Cirrus (Carl Zeiss Meditec) [19]. Both reports concluded that the choroid layer is thicker in the subfoveal area, and the nasal area is thinner than the temporal one as well. The results obtained with Spectralis showed a central choroidal thickness of 287 ± 76 µm (n = 30) and 272 ± 81 µm with Cirrus (n = 34) [18, 19]. In our study, according to the findings of Margolis and Manjunath, central or subfoveal thickness in global analysis was the thickest area in this layer, and this measurement was confirmed in age and gender subgroup analysis (263 ± 48 um).

A comparison of our central choroidal thickness results with the literature revealed a thinner central choroid in the Hispanic study sample, a novel finding with high relevance as a new parameter for ocular and cerebral vascular disease evaluation. Nasal and temporal choroidal thicknesses did not show a statistically significant difference in our results.

## Conclusions

Finally, our study shared the first normal value database to measure macular and choroidal thicknesses in the Hispanic population using Optovue. This novel dataset will allow a more objective and precise comparison between Hispanic patients in global analysis and adjusted for age and gender, in contrast to values reported previously by manufacturers or clinicians with these technologies based on other ethnic groups. The limitations of the study are mainly related to difficulty reaching the estimated sample size (n = 369) and an imbalance of gender participation including more women than men. Regarding age stratification, Group 1 included more subjects than Group 2 and 3, a risk for selection bias. To the best of our knowledge, this is the first report in the Hispanic population regarding normal cut-off values of retina and choroid thickness using SD-OCT Optovue. These new data give accurate parameters in Hispanics to rule in or out clinical diagnosis regarding posterior segment diseases.

## Data Availability

The datasets used and/or analyzed during the current study are available from the corresponding author on reasonable request.

## Abbreviations

CI: Confidence interval
IQR: Interquartile range
OCT: Optical coherence tomography
RPE: Retinal pigment epithelium
SD: Standard deviation
SD OCT: Spectral domain optical coherence tomography
SMD: Standard median deviation
TD: Time domain
